# Neuronal Spike Train Analysis in Likelihood Space

**DOI:** 10.1371/journal.pone.0021256

**Published:** 2011-06-27

**Authors:** Yousef Salimpour, Hamid Soltanian-Zadeh, Sina Salehi, Nazli Emadi, Mehdi Abouzari

**Affiliations:** 1 School of Cognitive Sciences (SCS), Institute for Research in Fundamental Sciences (IPM), Tehran, Iran; 2 Department of Biomedical Engineering, Johns Hopkins School of Medicine, Baltimore, Maryland, United States of America; 3 Center of Excellence for Control and Intelligent Processing, Department of Electrical and Computer Engineering, University of Tehran, Tehran, Iran; 4 Image Analysis Laboratory, Department of Radiology, Henry Ford Health System, Detroit, Michigan, United States of America; 5 Research Group for Brain and Cognitive Sciences, Shahid Beheshti University of Medical Sciences, Tehran, Iran; Tel Aviv University, Israel

## Abstract

**Background:**

Conventional methods for spike train analysis are predominantly based on the rate function. Additionally, many experiments have utilized a temporal coding mechanism. Several techniques have been used for analyzing these two sources of information separately, but using both sources in a single framework remains a challenging problem. Here, an innovative technique is proposed for spike train analysis that considers both rate and temporal information.

**Methodology/Principal Findings:**

Point process modeling approach is used to estimate the stimulus conditional distribution, based on observation of repeated trials. The extended Kalman filter is applied for estimation of the parameters in a parametric model. The marked point process strategy is used in order to extend this model from a single neuron to an entire neuronal population. Each spike train is transformed into a binary vector and then projected from the observation space onto the likelihood space. This projection generates a newly structured space that integrates temporal and rate information, thus improving performance of distribution-based classifiers. In this space, the stimulus-specific information is used as a distance metric between two stimuli. To illustrate the advantages of the proposed technique, spiking activity of inferior temporal cortex neurons in the macaque monkey are analyzed in both the observation and likelihood spaces. Based on goodness-of-fit, performance of the estimation method is demonstrated and the results are subsequently compared with the firing rate-based framework.

**Conclusions/Significance:**

From both rate and temporal information integration and improvement in the neural discrimination of stimuli, it may be concluded that the likelihood space generates a more accurate representation of stimulus space. Further, an understanding of the neuronal mechanism devoted to visual object categorization may be addressed in this framework as well.

## Introduction

Establishing a quantitative correlation between neuronal spiking activity and an external stimulus is a challenging task in neuroscience. It is known that neurons generate series of spikes in response to the stimulus. Each spike train is a stochastic process composed of a sequence of binary events that occurs in continuous time [1]. The point process theory is used as a stochastic framework to model the non-deterministic properties of the neural spike trains, in which its parameters are estimated by recording the spike trains of a neuron in repeated trials [2]. Such point process models can capture most of the nonlinear and stochastic properties of the neurons such as dynamic stimulus modulated responses [3].

The state space point process filtering approach is commonly used to model neuronal spiking activity [3], [4]. This framework allows for dynamic modeling, an important tool in computational neuroscience for studying neural stochastic behaviour [5]. Aspects of neuronal dynamic include neural receptive field plasticity [6], [7], neural coding analyses [8], [9], neural spike train decoding [10], [11], neural prostheses [12], [13], analyses of learning [14], [15], analysis of neuronal spiking dynamic [16], and control algorithm design for brain-machine interfaces [17], [18]. In most conventional methods, the neuronal firing rates of spiking activity are considered as a source of information and the temporal information is not included in the processing algorithms [19], [20]. In the use of temporal analysis in encoding stimulus information, the neuronal rate functions are typically not considered [21]. However, some experiments do show different kinds of integration in temporal and rate information in encoding the stimulus features [22].

Many neuroscience experiments, aim to investigate how dynamic properties of neuronal systems, either at the single or population level, lead to the functional properties of specific brain regions [16]. The dynamic property of the neural system as a whole, especially in spike train recording, indicates the need for dynamic signal processing methods. Despite the development of efficient dynamic signal processing algorithms, most current methods for neural spike train data processing are static and rate function based rather than dynamic and temporal based. For this reason, there is an increased drive to develop dynamic signal processing methods explicitly for neural spike trains [23]. In this study, a new feature space is generated by considering spike trains as binary vectors and projecting them onto the likelihood space. In this space, we are able to integrate temporal and rate information and compensate for errors of modeling stimulus distribution in the observation space. These modifications may improve performance of distribution-based classifiers by transforming the decision region into a contiguous region in the likelihood space.

In this paper, we will first review point process modeling of neurons in terms of a conditional intensity function, and introduce the state space point process filtering approach through description of the parameter estimation method. Then, we will show that the likelihood function of a spike train can be estimated based on the proposed model, and that the likelihood space for each neuron may be generated by projecting its spike train. The marked point process will be used for extending the model from a single neuron to a population of neurons. Properties of the likelihood space for spike trains will also be investigated. A new interpretation for information content of a spike train regarding a specific stimulus will be introduced and used as a metric between the clusters of points in the projected space. These point clusters are therefore associated with the presented stimulus. Finally, we will demonstrate the efficiency of the estimation technique based on a goodness-of-fit criterion, and demonstrate properties of the likelihood space. This is accomplished through modeling of the neuronal spiking activity of the inferior temporal (IT) cortex in a macaque monkey performing a passive fixation task, both at single and population levels and illustration of neuronal representation of the visual stimulus space.

## Materials and Methods

### Point process modeling of a neuron

A stochastic neural point process can be completely characterized by its conditional intensity function. The conditional intensity function is a strictly positive function that gives a history-dependent generalization of the rate function of a Poisson process [24]. We use the conditional intensity function to characterize the spike train as a point process. We assume that in an interval 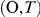 spikes are fired by a single neuron at times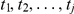 for 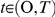. The conditional intensity function is defined as:
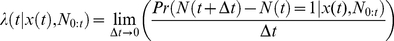
(1)


Where 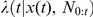 is a conditional probability, 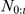 includes the neuron's spiking history and the trace of spikes occurrences up to time 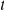, and 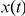 is a parameter to be estimated. 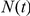 is the number of spikes fired by the neuron in 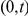. Because the conditional intensity function completely defines the point process, to model the neural spike train in terms of a point process suffices to define its conditional intensity function. Parametric models may be used to express the conditional intensity as a function of covariates of interest [24].

In order to represent the point process model, we discretize the time interval 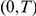 by dividing it into 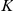 intervals of width 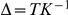 such that there is at most one spike per interval. For 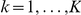, let 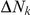 be the indicator of a spike in the interval 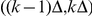, which is one if there is a spike and zero otherwise. We let 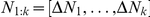 denote the spiking activity and 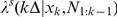 denote the conditional intensity function for the repeated trials when stimulus 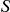 is presented. The likelihood of a neural spike train is defined by finding the joint probability density of the data. It is shown that the joint probability of any point process is derived from the conditional intensity function by considering it to be a product of conditionally independent Bernoulli events [24]. If again we assume that on an interval 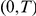, 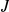 spikes are fired by a single neuron at times 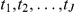 for the stimulus 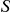, then the probability density of these 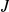 spikes in 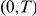 is:
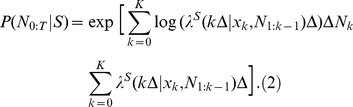
(2)


We can evaluate the likelihood that the spike train comes from stimulus 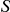 by calculating the value of 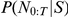 using Equation (2). In this evaluation, we use the temporal pattern of spike train weighted by conditional intensity function [25]. In the rest of this paper, we use the marked point process to generalize the Equation (2) from single neuron to the population level.

### Projection of spike trains onto the likelihood space

If there are 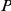 stimuli, any observed spike train 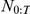 must be related to one of the 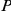 stimuli 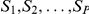. Let 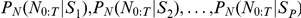 represent the true distributions of the spike trains from the 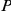 stimuli. Let 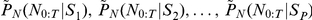 represent estimates of the true distributions. The likelihood projection of a sample path of spike trains is defined as the operation 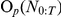 resulting in a *P*-dimensional likelihood vector, 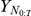, as in Equation (3). 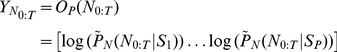
(3)


**Figure 1:**
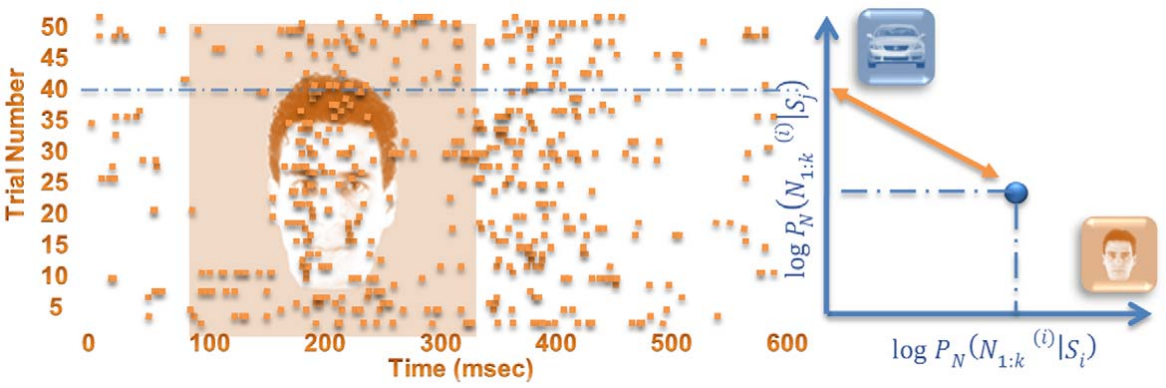
Projection of spike train onto likelihood space. Sample response of a single neuron to face stimulus presentation in raster plot format. This data is for the repeated trials, where each row is the spike train recorded for any individual trial. The transformation of the spike train for the single trial, from the observation space into a likelihood space, is illustrated. Based on previous observations and estimated stimuli conditional probability distribution, each point in the new space is generated by the projection of the binary vector of spike train.

The distributions 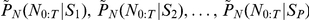 are the projecting distributions and the *P*-dimensional space whose coordinates are 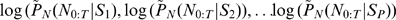 is the likelihood space: When the dimension of the observation vector 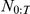 is greater than 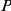, the likelihood projection operation 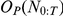 is a dimensionality reducing operation (Figure 1) [26].

### Properties of spike trains in the likelihood space

By constructing the likelihood space from the spiking activity of the neurons, clustering the projected neural data, and decoding the stimulus from the spike train, a categorization of stimulus can be achieved. This can be considered as a distribution-based classification problem. Likelihood vector representations have several properties that relate to clustering and classification in the likelihood space, which we describe below.

First, each spike train is assumed as a binary vector that contains temporal information in addition to the rate information. If for instance on an interval 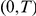, 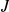 spikes are fired by a single neuron at times 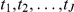 for the stimulus 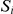, with conditional intensity function 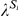, we can reinterpret Equation (2) as an indication of the dependency of the components in the likelihood vector to temporal arrangement of the spikes, which in turn is weighted by the value of the conditional intensity.

Second, the projecting distributions represent a set of decision boundaries in the observation space that partition it into 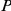 decision regions. The decision region 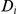 for stimulus 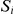 is the region defined by:
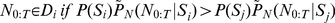
(4)where 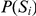 represents the a priori probability of stimulus 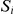. The decision regions defined by Equation (4) may consist of several disconnected regions [26]. In the likelihood space, these regions are projected onto a region 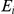 defined by:
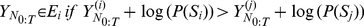
(5)


Equation (5) shows that if 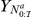 and 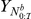 both lie within 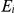 then 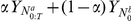 lies in 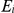 for any 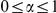, thereby proving that the region 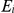 is convex and therefore connected.

Finally, in the observation space, the optimal minimum-error Bayesian classifier is given by the rule that 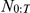 is classified as belonging to the stimulus 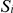, such that 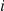 indexes the stimulus with the largest value for 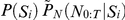. [23]. A classifier that uses estimated distributions can be equivalently stated in terms of log-likelihoods as 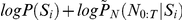. Classification between any two stimuli 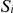 and 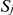 is done as shown in Equation (6). By considering 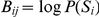 and 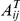 a vector of 1 in the 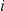 component and -1 in the 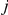 component and 0 in the other components, Equation (6) can be redefined in the likelihood space as Equation (7), which is a simple linear discriminant with a slope of unity. 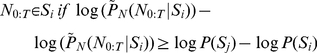
(6)

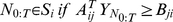
(7)


It is thus possible to define a classifier in the likelihood space that performs identically to a Bayesian classifier based on the projecting distributions in the observation space. It follows that the performance of the optimal classifier in the likelihood space cannot be worse than that in the observation space. It also follows that if the projecting distributions are the true distributions of the stimulus, then the optimal classification performance in the likelihood space is identical to the optimal classification performance in the observation space [26].

### Extended Kalman filtering of a point process

The state space point process filtering approach is used for optimal estimation of parameters. In this approach, the counting process 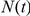 is used by an observation equation as:
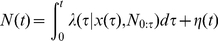
(8)where 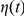 is a zero mean error process that is the residual between a point process and its expectation. We construct a discrete time version of the observation Equation (8) for a fine partition of the observation interval, linearize its expected value as a function of the state process by using the linear terms of a Taylor expansion about the one-step prediction mean, and add Gaussian white noise errors as Equation (9).
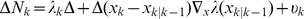
(9)


In Equation (9), 
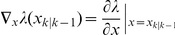
 and the Gaussian error term 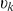 should be selected so as to have similar statistical properties of the observation distribution. The variance of the discrete time approximation to the point process model is 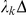, which is unknown. Since 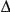 is sufficiently small the 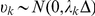 might be a good choice. The state equation in Equation (10) is the Gaussian linear stochastic system where 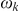 is a zero-mean Gaussian noise with covariance matrix 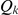.
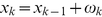
(10)


We model the conditional intensity function in terms of the state process as
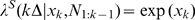
(11)


In this kind of modeling, the history dependency in spiking activity within a trial is defined in terms of a state process and the spiking activity between trials is independent. The exponential function is used as a parametric model for conditional intensity to ensure that the 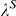 is strictly positive [24].

It follows from the theory of point processes that by taking the discrete approximation of the joint probability density of the spike train on the specific interval 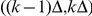, the probability mass function of the observation equation for our state-space model is defined as:
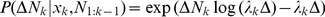
(12)


We define 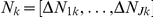 as all the observation in the interval 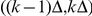 across all 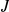 trials, 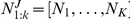 is the matrix of all observation across the trials and 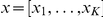 is unobservable state vector.

To evaluate the neuronal response to specific stimulus, as related to the model in Equation (11), we apply the maximum a posteriori derivation of Kalman filter. We further approximate 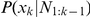 as Gaussian probability densities by recursively computing their means and covariance matrices. For initiating the recursive algorithm, let 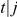 denote the expectation of the state variable at 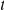 given the responses up to time 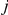. We assume that the mean 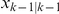 and covariance matrix 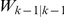 have been estimated at time 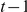. That is, we take 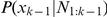, the posterior probability density at time 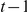, to be the Gaussian probability density with mean 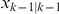 and covariance matrix 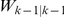. The next step is to compute 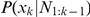, the one step prediction probability density at time 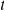. This is the probability density of the predicted response at 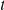 given the spiking activity in 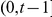. It follows from standard properties of integrals of Gaussian functions, and the state equation in Equation (10), that the mean and covariance matrix are defined as

Predicted state
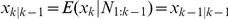



Predicted estimated covariance
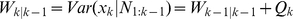
(13)which correspond, respectively, to the one-step prediction estimate and the one-step prediction variance.

Innovation or measurement residual
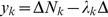
(14)


Innovation or residual covariance
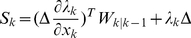
(15)


Optimal Kalman gain
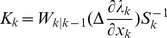
(16)


Update state estimate
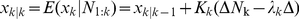
(17)


Update estimate covariance
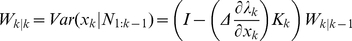
(18)


The Equation (18) can be rewrite as follows
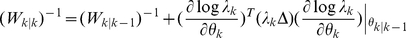
(19)


In this way, the Kalman filter recursive equations are completely derived for point process observation of the neuronal spiking activities [27].

### Marked point process modeling of a population

We consider a population of 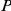 neurons responding simultaneously to a presentation of a stimulus. Their responses are denoted by a vector 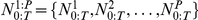 where 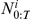 represents the stochastic response of the 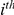 neuron to a stimulus. The stimulus state is denoted by the scalar variable 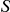, which is discrete in our case and selected with uniform probability from a stimulus set.

In order to find the probabilistic model for the populations of neurons, we apply the marked point process theory. Let 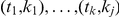 be the observation of 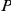 neuron over the interval 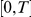. The 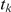 is the spike instant in the pooled trains and the 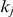 is the label of the neuron which fires at time 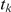 [24], [28]. The log likelihood function 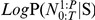 of such a realization may be expressed in the form of the marked point process.
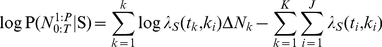
(20)


In this assumption, the marked point process is the combination of two independent processes; the ground process and the marked process. The ground process is the result of pooling all the spikes in the interval 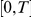, and the marked process is the result of observing the label of the fired neuron at any spike instant. The conditional intensity function of population 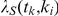 can be written as Equation (21) where 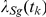 is the intensity of the ground process, and 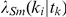 is the intensity of a mark process at given time 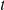.
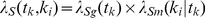
(21)


The conditional intensity of the ground process is modeled with the summed intensities of the neurons in the ensemble. This can be found in Equation (22). The mark process that determines to which neuron the spike time should be attributed is randomly sampled for each spike time. This sampling comes from a multinomial distribution with probability parameters as indicated in Equation (23)
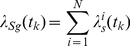
(22)

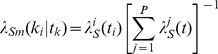
(23)


By inserting the Equations (22) and (23) in (21) and substituting Equation (21) in (20), the Log-Likelihood function for a marked point process model of 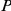 neurons in the population, while the neurons are observing the stimulus 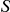 can be written as Equation (24) [24], [28].
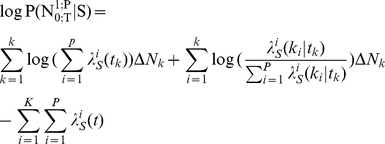
(24)


The Equation (24) is an extension of Equation (2) and can estimate the probability of observing response vector 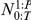 for the populations of neurons.

### Information theoretic interpretation of spike trains in the likelihood space

Consider a neuronal population presented with an ensemble of stimuli, called 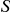. Their behaviour can be represented with a set of responses, represented as 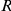. The mutual information between the stimulus 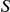 and response 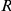 of this system is given by
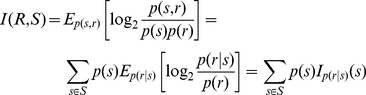
(25)where the 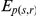 and 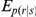 are the expectations with respect to the 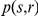 and 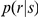. The 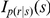 is the information specifically conveyed about each stimulus. It is a direct quantification of the variability in the response elicited by the stimulus, compared to the overall variability [29].

Suppose 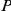 neurons are responding to the stimulus set 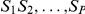 with the distributions of the spike trains 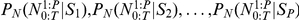 as shown in Equation (24). For any set of observations 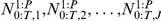 while the stimulus 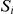 was presented, we can consider the spike trains as a binary vector and project them onto the likelihood space (Equation (3)). By scaling each component with the probability distribution of responses averaged across stimuli 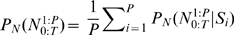, we can write the expectation of the projected vector with respect to 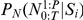 as:
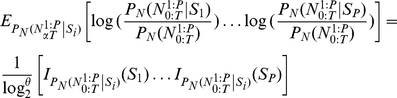
(26)


Each component of expectation vector is equal to the
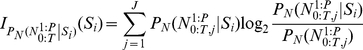
(27)where 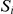 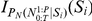 is the information specifically conveyed about stimulus 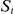 by the population. By projecting all observations onto the likelihood space and scaling each component to the average response, we can define the distance between two stimuli 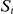 and 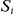. This definition is with respect to the spiking activity of the 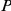 neurons while each of them is presented, as follows:
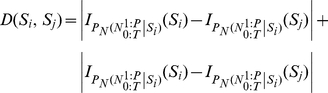
(28)


The 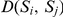 is a difference between the information specifically conveyed about the two stimuli based on point process observation of the population 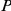 neurons [30].

### Goodness-of-fit tests

We use the time-rescaling theorem to construct a goodness-of-fit test for a neural spike data model. Given a point process with conditional intensity function 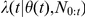 and occurrence times 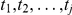 where 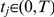, if we define 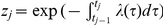, then these 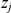 are independent, exponential random variables with rate parameter one [10]. A common approach to measuring agreement between the model and the data is to construct a Kolmogorov-Smirnov (KS) plot. The KS plot is a plot of the empirical cumulative distribution function (CDF) of the rescaled times against an exponential CDF. If the conditional intensity model accurately describes the observed spiking data, then the empirical and model CDFs should roughly coincide, and the KS plot should follow a 45° line. If the conditional intensity model fails to account for some aspect of the spiking behaviour, then that lack of fit will be reflected in the KS plot as a significant deviation from the 45° line. Confidence bounds for the degree of agreement between a model and the data may be constructed using the distribution of the KS statistic [31].

### Multidimensional scaling

Multidimensional scaling is a set of data analysis techniques that display the structure of distance-like data as a geometrical picture. Each object or event is represented by a point in a multidimensional space. The points are arranged in this space so that the distances between pairs of points reflect the similarities among the pairs of objects. This is to say that two similar objects are represented by two points that are close together, and two dissimilar objects are represented by two points that are far apart. A dissimilarity matrix must be real and symmetric with zeros along the diagonal and positive values elsewhere. In this paper, the classical multidimensional scaling is implemented by constructing a 2-dimensional space using the eigenvectors of the dissimilarity matrix corresponding to the two largest eigenvalues [32].

### Animal treatment and surgery

A male macaque monkey (*M. mulatta*) participated in the current study. All experimental procedures complied with the guidelines of the National Institutes of Health and the Iranian Society for Physiology. The use of non-human primates in this research was also in accordance with the recommendations of the Weatherall report, “the use of non-human primates in research”. The study protocol was approved by the ethics committee of School of Cognitive Sciences (SCS), Institute for Research in Fundamental Sciences (IPM) under permit number 08-06-83132001. In a preparatory aseptic surgery, a block for head fixation and a recording chamber was anchored to the dorsal surface of the skull. A craniotomy was performed and the position of the recording chamber was determined stereotaxically referring to the magnetic resonance images (MRIs) acquired prior to surgery. The animal was first tranquilized with 0.2 mg/kg of atropine (i.m.) followed by 5 mg/kg of ketamine (i.m.). For prolonged anaesthesia thereafter, a bolus of sodium pentobarbital (20 mg/kg) was injected intraperitoneally and was repeated if needed. Body temperature was maintained around 37°C with a regulated heating pad. Before the surgical procedure, a preventive dose of antibiotic (ceftriaxone 250 mg, i.m.) was administered. Antibiotic and analgesic (ceftriaxone 250 mg, i.m., b.i.d. and ketorolac 0.5 mg/kg, i.m., b.i.d.) were administered postoperatively for 4 days.

### Recordings and stimuli

**Figure 2:**
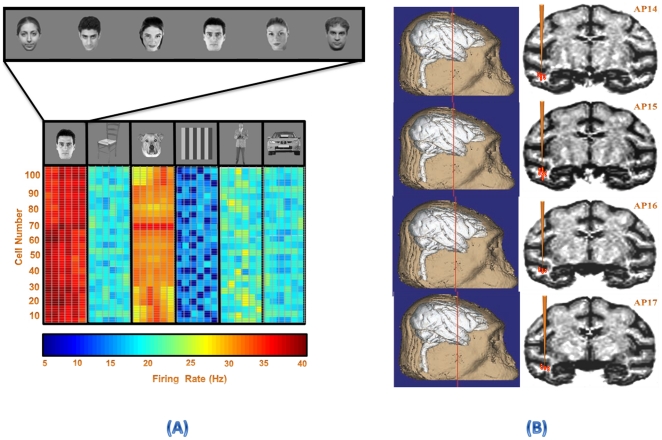
Recording areas and the average firing rate's response of the neuronal population. Recording positions were evenly distributed at anterior 14–20 mm over the ventral bank of the superior temporal sulcus and the ventral convexity up to the medial bank of the anterior middle temporal sulcus with 1-mm track intervals.

Following a two-week recovery period, action potentials of single cells were recorded extracellularly with tungsten electrodes (FHC, ME). These recordings were taken from the IT cortex while the monkey performed a fixation task with its head restrained. The recording positions were determined stereotaxically referring to both MRIs acquired before the surgery and the gray and white matter transitions determined during electrode advancement [33]. The electrode was advanced with an oil-driven manipulator (Narishige, Japan) from the dorsal surface of the brain, through a stainless steel guide tube inserted into the brain, down to 10–15 mm above the recording sites. Recording positions were evenly distributed at anterior 15–20 mm over the ventral bank of the superior temporal sulcus and the ventral convexity up to the medial bank of the anterior middle temporal sulcus with 1-mm track intervals as illustrated in Figure 2. The recording was not biased by response properties. The action potentials from a single neuron were isolated by an offline sorting algorithm (Plexon Inc.).

**Figure 3:**
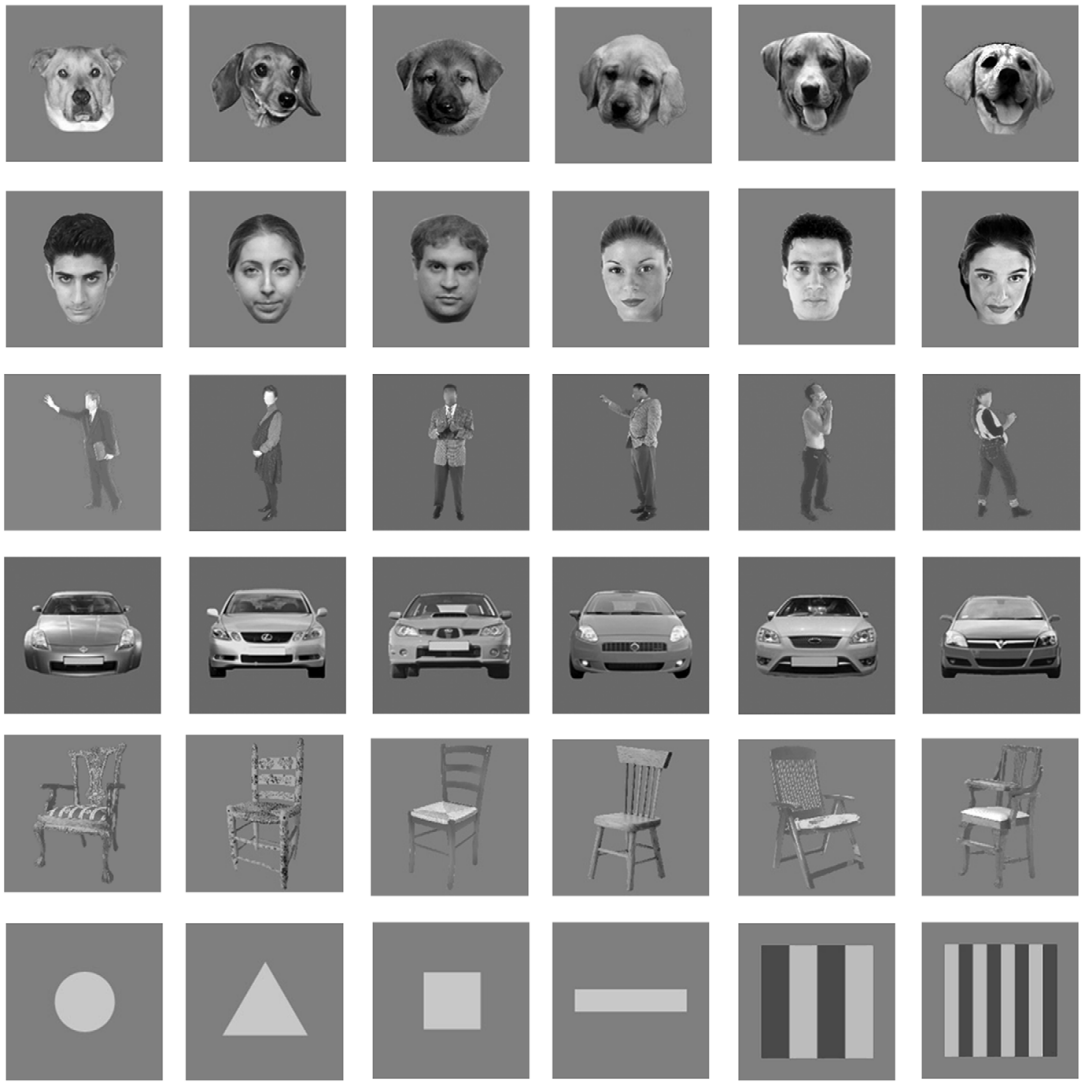
Visual stimulus set. A set of visual objects from six different categories with gray background was selected as stimuli while the monkey performed the passive fixation task. Human face, human body, dog face, car, chair, and simple shape were the selected categories for presentation.

Responses of each cell were recorded to stimuli presented in a pseudorandom order. The stimulus set was repeated 49±2 (median, 50) times for each recording site. The sequence of stimuli was changed randomly between different sets, and also between different recording sites, to avoid any consistent interaction between successively presented stimuli. The stimuli were 36 gray scale photographs of natural and artificial objects isolated on a gray background. The stimulus set consisted of six different categories (human face, human body, dog face, car, chair, and simple shape); each contained 6 number of identical member (Figure 3). The size of the larger dimension (vertical or horizontal) of each stimulus was ∼7° of visual angle.

**Figure 4:**
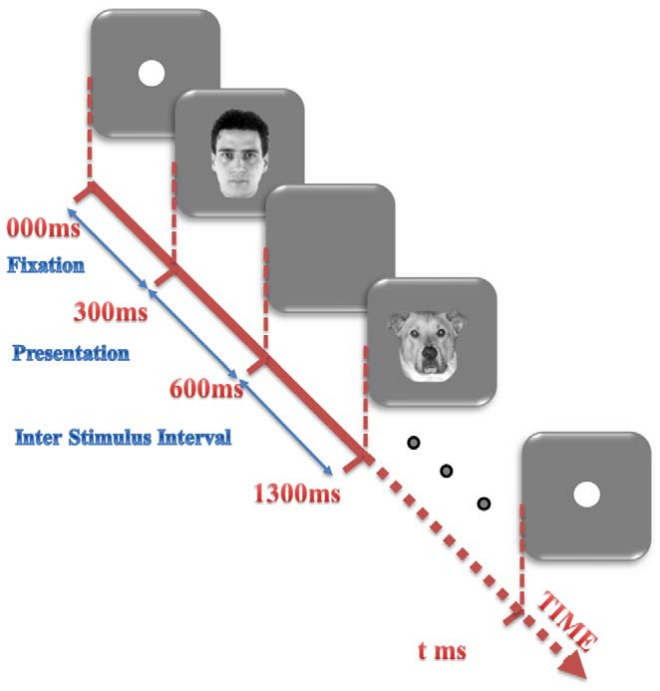
Passive fixation task. The paradigm for the passive fixation task is illustrated. The presentation of the stimulus sequence started after the monkey maintained fixation for 300 ms. Each stimulus lasted 300 ms and was followed by another stimulus after a 700 ms interstimulus interval. The sequence stopped when 36 stimuli were presented, or when the monkey broke the gaze fixation.

The monkey had to maintain fixation within ±2° of a 1° fixation spot presented at the center of the display. The eye position was measured by an infra-red eye-tracking system (i_ rec, http://staff.aist.go.jp/k.matsuda/eye/), which allowed a precision of 1 degree or less for the measurement of eye position [34]. Each block started with appearance of a 1° fixation point in the center of the display. When the monkey maintained his gaze at the fixation point for 300 ms, the fixation point disappeared and presentation of the stimulus sequence started. Each stimulus was presented for 300 ms, with a 700-ms interstimulus interval (Figure 4). The sequence stopped when 36 stimuli were presented or when the monkey broke the gaze fixation, and a new block started with the reappearance of a fixation point. The monkey was rewarded with a drop of fruit juice every 1–3 s during the fixation.

## Results

In order to illustrate some of the properties of the likelihood space, the neural data of spiking activity from the IT cortex neurons of a macaque monkey was used. Each stimulus was presented for 300 ms and followed by 700 ms interstimulus blank interval. A 100 ms interval before stimulus presentation was recorded for the purpose of baseline activity study. Category selective neurons were entered in this study with face selectivity as the most important feature for inclusion [34], [35]. Recording areas and the average firing rate's response of the neuronal population are illustrated in Figure 2.

### Point process modeling of the IT cortex neurons

**Figure 5:**
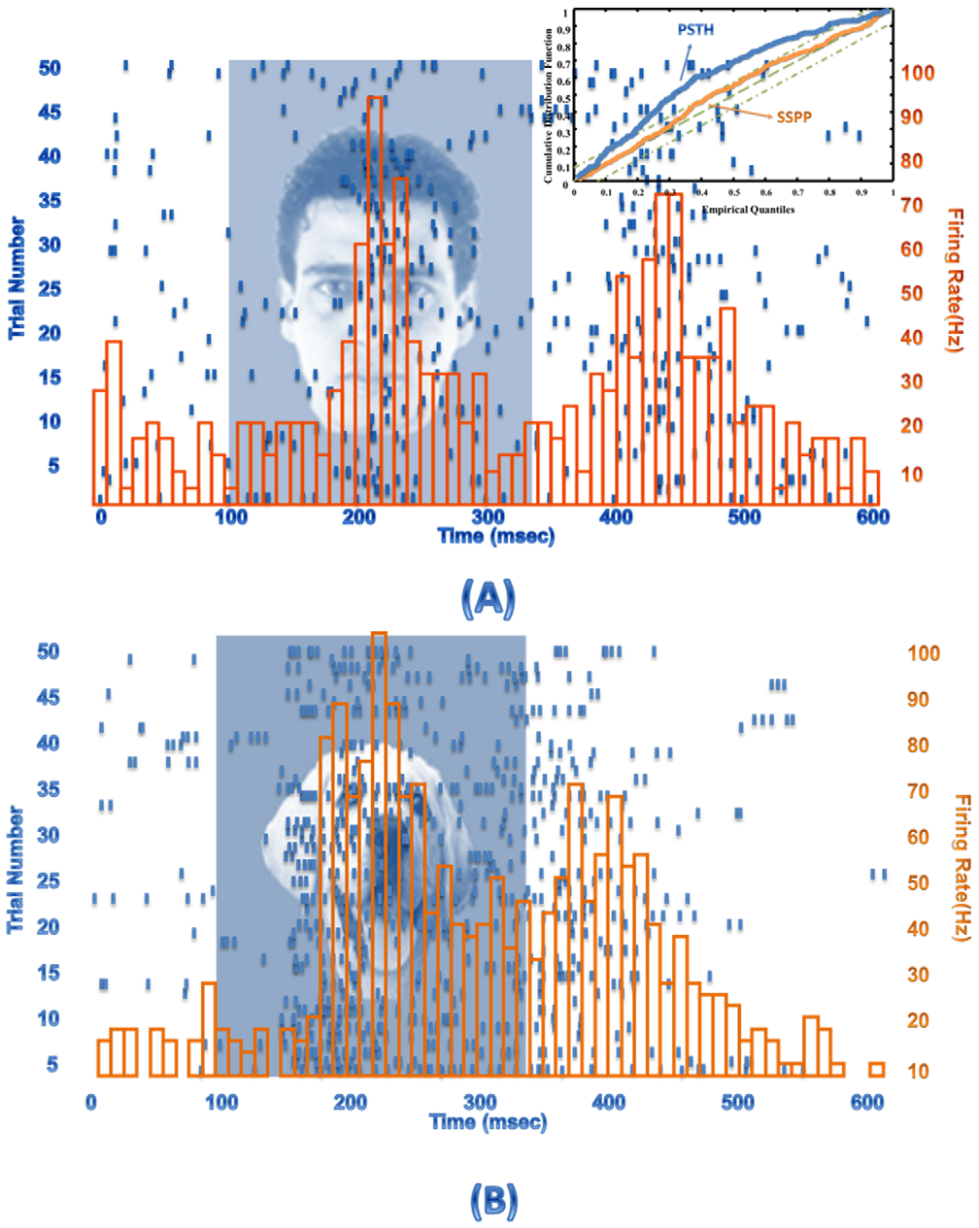
Model parameter estimation. Sample responses of a neuron from IT cortex of a macaque monkey while performing the passive fixation task. The spike trains in repeated trials, in the form of a raster plot and the estimated conditional intensity function are shown for (A) a human face presentation with 95% goodness-of-fit criteria and (B) a car presentation. For face stimulus the raster plot is used for fitting the point process model on the neuronal responses with the conditional intensity estimation. The goodness-of-fit criterion is used to compare the point process model with conventional peristimulus time histogram.

Based on the conditional intensity function model, point process filtering was applied and model parameters were optimally estimated. The stimulus effect in the conditional intensity model for the visual object was optimally estimated with 95% goodness-of-fit criteria for face stimulus as shown in Figure 5. Based on the goodness-of-fit criterion the point process model can capture the conditional intensity more accurately with respect to the conventional peristimulus time histogram (PSTH) as illustrated in Figure 5(A) top right corner. The conditional intensity was used for the likelihood function estimation for each stimulus.

### Likelihood space generation for visual objects

**Figure 6:**
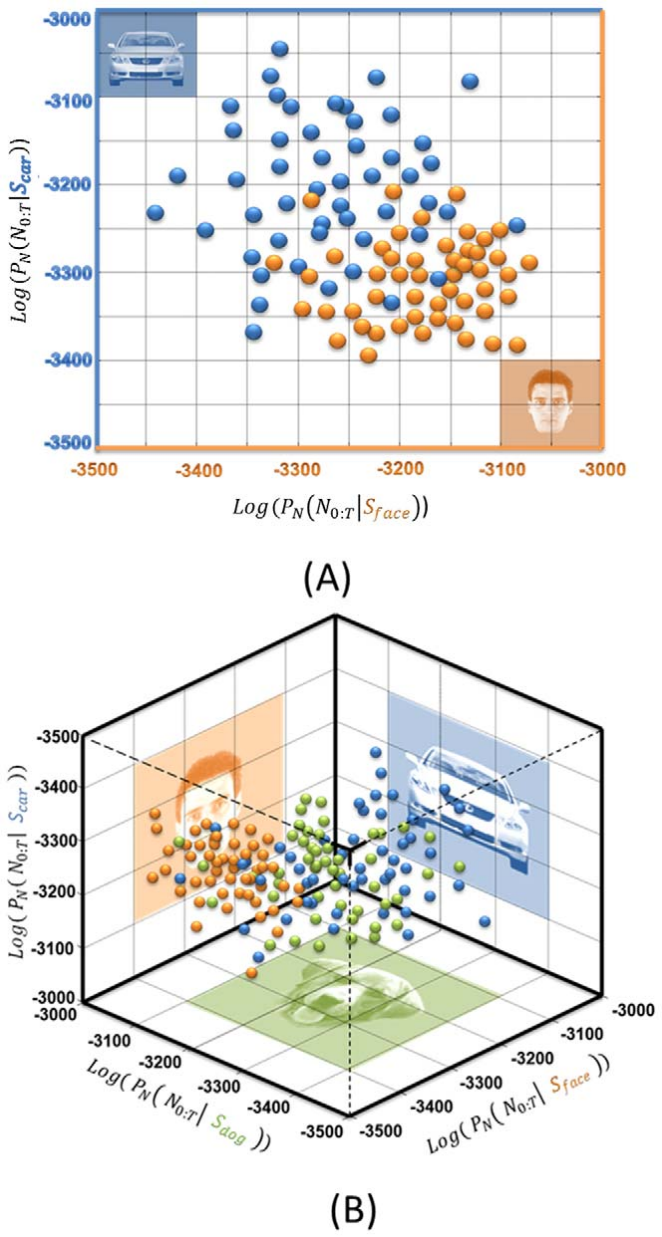
Projection onto likelihood space. The repeated trial observation of neuronal spiking activity was used to estimate the probability model of the spike train. This enabled us to transfer any spike train into likelihood space and represent it as a single point. The coordinate components of this point are equal to the probability of spike train generated from a specific stimulus. (A) Reconstruction of likelihood space for the neural activity of a single neuron in IT cortex, while the human face and car pictures were presented. Since we reconstructed the space with respect only two stimuli, the projected space has only two dimensions. (B) The likelihood space was generated for the same neuron while spike trains from presenting human face, dog face, and car images were projected.

The spike trains of the face selective neurons were projected onto the likelihood space (Figure 6). The components of the projection were estimated based on Equation (2). The dimension of the likelihood space was equal to the number of the stimuli; it can be created for any combination of stimulus sets. Figure 6(A) shows the projection of the neural activity in IT cortex when the human face and car images were presented to the monkey. In Figure 6(B), a three-dimensional likelihood space is shown for the presentations of human face, dog face, and car to the same neuron.

### Properties of spike train projection

**Figure 7:**
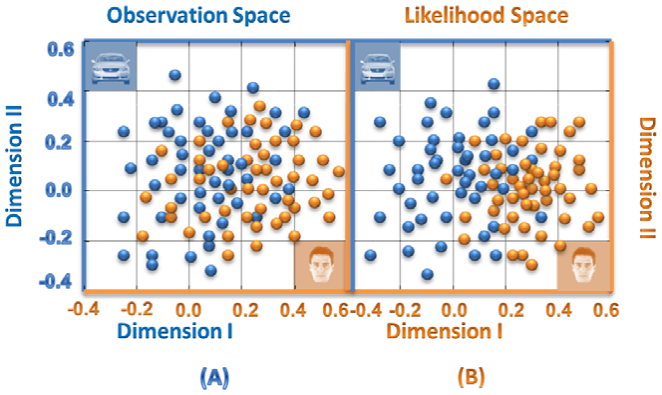
Multidimensional scaling in observation space and likelihood space. A multidimensional scaling technique is used to illustrate the capability of the likelihood space in increasing the separability of the clusters. (A) The distance measurement and multidimensional scaling results for pairs of spike trains from the human face and car stimuli in the observation space. (B) The distance measurement and multidimensional scaling results for the same spike trains after projection onto the likelihood space.

In order to evaluate the “closeness” of the spike trains stimulus after projecting them onto the likelihood space, the multidimensional scaling technique was applied to pair-wise comparisons of the entities. The multidimensional scaling allowed us to visualize closeness of spike trains by representing them in a low-dimensional space [33]. The amplitude of the difference between any pairs of spike train vectors is defined as a distance in the observation space. The results of the multidimensional scaling analysis on normalized proximity matrices are shown in Figure 7. Figure 7(A) is an illustration of the two dimensional representation of the pair-wise distances between spike train vectors before projection. Figure 7(B) shows the result of analysis on the distance measure in the likelihood space between any pairs of projected points and two dimensional representation based on the multidimensional scaling analysis. The Fisher's discriminant ratio was used to quantify the separability of the clusters in the two spaces. This criterion showed an average of approximately 26% improvement in the separability of clusters in the likelihood space. This result indicates that the clusters are more separable in the likelihood space.

### Generation of the likelihood space for the populations of the IT neurons

The projected points represent each stimulus as a cluster. The clusters can be considered as estimates representing the populations of neurons from the stimulus space. We used the distance between the center of the clusters as a neuronal representation of similarity. The closer cluster represents similar visual objects. The accuracy of the representation depends on the efficiency of the estimation method, and the number of the spike trains observed.

**Figure 8:**
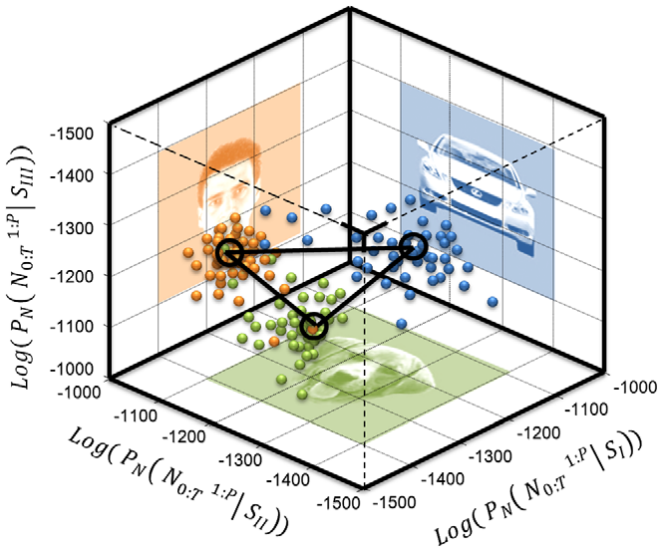
Extending the likelihood space for populations of neurons. The likelihood space generation for populations of neurons based on projecting the spiking activity of the population recorded from 100 neurons in IT cortex. These recordings were taken while the human face, dogface, and car images were presented to the monkey. The marked point processes theory was used for developing the probability model for the population.

We used the neural response of 100 neurons recorded from the IT cortex of the monkey while doing the previously described passive fixation task [35]. The spike trains of the neural ensemble in response to the human faces, cars, and dog faces for 50 repeated trials in 70–270 ms time intervals were modeled using marked point process and projected onto the likelihood space. These are shown in Figure 8. By scaling each component with the response average and estimating the expectation of the component in the log-likelihood space, we estimated the stimulus specific information based on the center of each cluster. The centers of the clusters were used for representing each stimulus category. The relative geometrical location of the cluster is considered as an interpretation of the neuronal population from the observed stimulus set.

### Visual object specific information estimation

**Figure 9:**
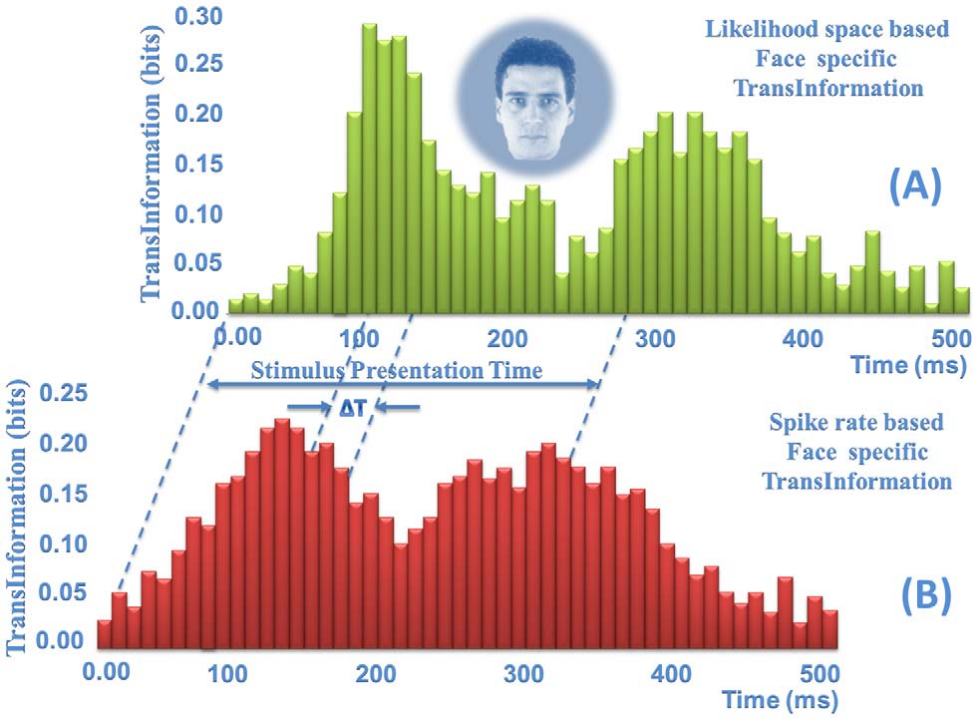
Information content of the face neuron. (A) The face specific information is approximated in the rate based framework. Based on a peristimulus time histogram, the relevant probability densities are estimated empirically and used for face specific information calculation. (B) The face specific information is approximated in the likelihood space based framework. The probability model of the joint spiking activity is used for face specific information estimation.

The encoding of information by face selective neurons was analysed using a quantitative information theoretic approach. We attempted to estimate the stimulus specific information based on Equation (27). The face specific information was approximated in the rate based framework. Based on a peristimulus time histogram, the relevant probability densities were estimated empirically and used for face specific information calculation. The same spiking data was used for the face specific information approximation in the likelihood space based framework. Instead of using the empirical probability density estimation, the probability model of the joint spiking activity was used for estimating the probability of spike train in any given time interval. In Figure 9, the amount of information about a specific face for a face selective neuron is estimated in two frameworks. In order to compare the amount of information and the temporal dynamic of its transmission, we used a 100-ms sliding windows with 10-ms steps. As shown in Figure 9, there are differences not only with respect to the quantitative value of the information, but also in temporal dynamic of the face information transmission.

### Visual object representation in likelihood space

**Figure 10:**
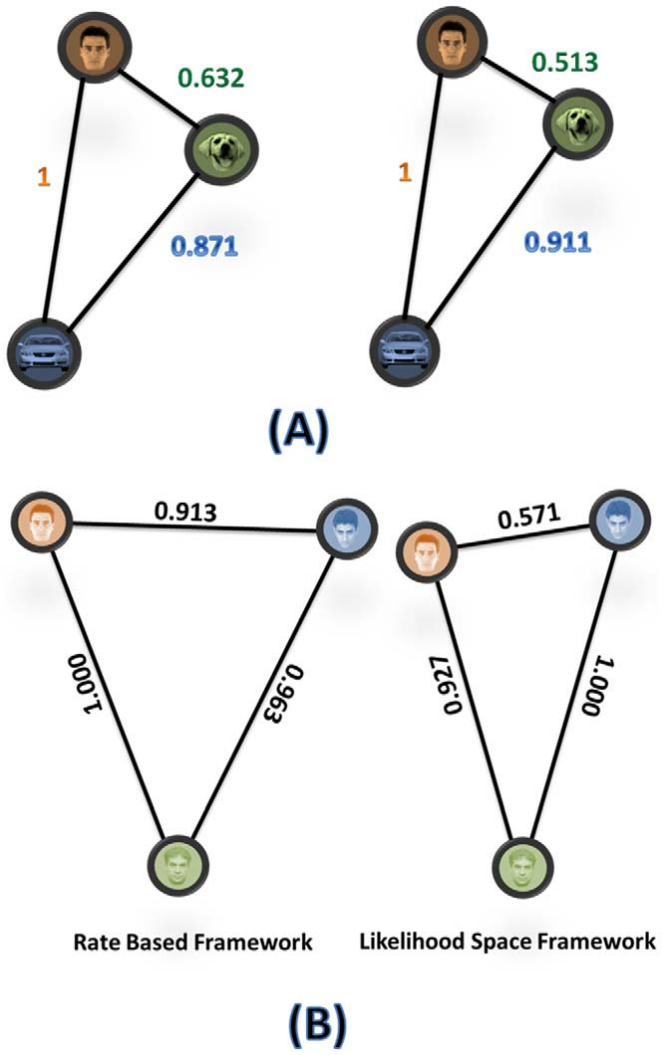
Between-stimulus distance measure. The likelihood space and correlation based representations of stimulus space for the populations of neurons while presenting human face, dog face and car images. The normalized neural representation of distance in the correlation based (A) and likelihood space (B).

In order to have a better comparison between the rate-based framework and temporal-based analysis, we use the neuronal activity of the same population in the same interval. We estimated the average firing rates of any individual neuron in the 70–270 ms time interval and arranged them in a vector. The vectors were normalized and divided by their Euclidean lengths. We calculated a correlation-based distance measure and constructed a relative geometrical interpretation of the different categories [34], [36]. In Figure 10, the normalized distance measures, based on similarity in the rate-based framework and the likelihood space framework, are shown. As illustrated in Figure 10 (A), when the three stimuli were chosen from different categories the normalized representations in the likelihood space was similar to the rate based framework but in the case of three faces from face category the normalized distance in two frameworks were different (Figure 10 (B)).

### Dynamic of the distance representation

**Figure 11:**
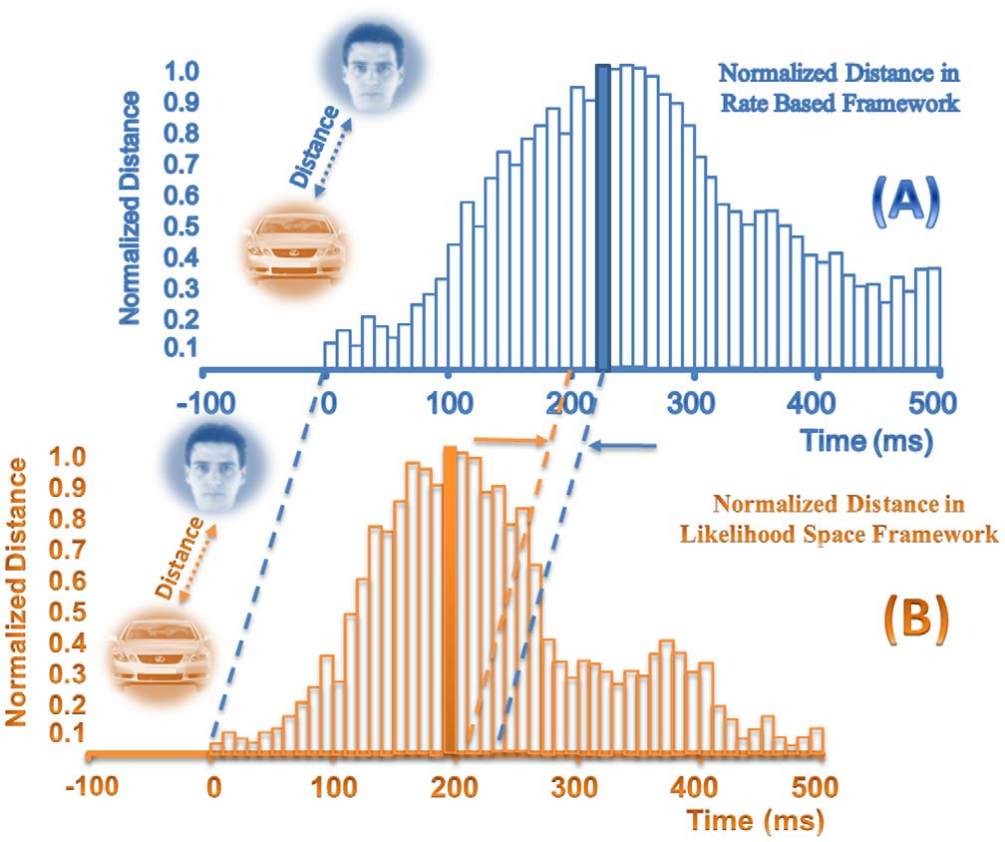
Dynamic between-stimulus distance measure. (A) Dynamic distance measurement between pairs of stimuli from two different categories in a 100-ms sliding time window, with 10-ms sliding step based on correlation distance. (B) Dynamic distance measurement for the same stimulus pair with 100-ms sliding time window and 10-ms sliding step based on stimulus distance in the likelihood space.

Based on the distance measurement and similarity, we used another analysis to compare the two frameworks. We applied a 100-ms sliding time window with a step size of 10-ms and found the distance between two different categories in each step. In Figure 11, the distance or dissimilarity between human face and car categories was estimated in 100-ms sliding time window with 10-ms step size for the rate-based and the likelihood-space-based frameworks. We marked times of stimulus presentation and maximum distance occurrence in both frameworks. We used latency of maximum dissimilarity occurrences as a criterion for temporal analysis of maximum information transmission.

## Discussion

In this research, a new approach for analysis of spike trains is introduced where each spike train is considered as a binary vector and projected onto a lower-dimensional space. Many covariates are sources of spike generation in a single neuron, and the observed spike trains are variable. The Kalman filtering based point process modeling approach, and the state space generalized linear model, help us to optimally estimate the conditional intensity function of the point process associated with each neuron for any stimulus. The time-rescaling theorem is used to construct goodness-of-fit tests for a neural spike data model. We model the spiking activity of the population of neurons using a single marked point process. This marked point process has a conditional intensity, which is the sum of the conditional intensities of all neurons in the population. To this end, class conditional distributions of stimuli are estimated and each observed vector is projected onto a specific point in the likelihood space.

The likelihood based approaches, which use the probability of neuronal response to a given stimulus, are widely used for fitting models and assessing their validity [37], [38]. They can be derived for several types of neural models and used for optimal decoding [39], [40]. In this study, we use the likelihood function to project spiking activity of neurons onto a new space, which may be a unique application of the likelihood-based approach in spike train analysis. This is a new probabilistic interpretation of the spike train that enables us to apply advanced signal processing and pattern recognition methods on neuronal data, at the single neuron and population levels.

Projections of spike trains onto the likelihood space have important advantages. First, since each spike train or observed vector is directly used in the projection process, the temporal information ignored in the conventional methods is considered here. Secondly, the projected vectors are more separable in the likelihood space, and also are less dependent on the accuracy of estimates of class conditional distributions. In this way they may improve the performance of distribution-based classifiers. Finally, since the coordinates of the likelihood space are the stimuli conditional likelihood and the numbers of stimuli are less than the dimensions of the spike trains' binary vector, the projection is a dimension reducing process.

The information theoretic approach has a number of important advantages that make it well suited for demonstrating the modulation of neural response by the stimulus [41]-[44]. We extend the use of information theory to analyze spike trains by modeling the joint probability density function between the ensemble spiking activity and the biological signal explicitly. We further compute stimulus specific information directly from the probability density function. In this approach, the direct parametric estimation of the conditional probability is used for the information estimation, which may be more accurate than rate-based approaches (Figure 9). We introduce a novel interpretation of stimulus specific information conveyed by a neuronal population. We show that the expectation of each component in the likelihood space is proportional to the amount of information it conveys about a specific stimulus by the population. Therefore, the difference between information content of the population, about a specific stimulus, can be considered as a distance metric and used for similarity measurement.

The limitations of the current study are: 1) the need for more observations compared to conventional methods; 2) the dependency of the model's accuracy in the population level on simultaneous observation of the neurons; and 3) the introduction of complicated mathematics with a higher computational load compared to conventional rate based spike train analysis, such as peri-stimulus time histogram.

While this study establishes the feasibility of constructing likelihood space for the neuronal populations as a linear stochastic dynamical system with point process observation models, several extensions for the current framework are possible. First, there is a possibility of extending the current algorithm to a nonlinear state space model for computing smooth estimation of state estimate [45]–[47]. Secondly, more biophysically realistic models can be used, which are based on a linear filtering stage followed by a noisy leaky integrate-and-fire spike generation mechanism [37], [38]. Thirdly, in our marked point process modeling of the population, we assumed that the neurons were independent given the value of the state process. Consideration of the possible functional dependency among neurons could broaden the application of the current framework to the various classes of the neuroscience problems. Fourthly, the emergence of multi-electrode arrays and the recent progress in multi-electrode recording enable us to interface with various populations of neurons simultaneously [48], [49]. The marked point process modeling and likelihood space representation of the population might be applicable in real time observations, as in the use of neuro-prosthetic devices [50], [51]. Fifthly, a novel extension of the use of information theory to analyze multiple spike trains from developing probability models of joint spiking activity might be useful for investigating behavior of neuronal populations in dynamic stimulus coding. Finally, by collecting enough observations from the neuronal population, the representation of the population from the stimulus space may be demonstrated. Problems such as determining the neural mechanism of stimulus categorization can be addressed in this framework.

## References

[1] Brown EN, Kass RE, Mitra PP (2004). Multiple neural spike train data analysis: state-of-the-art and future challenges.. Nat Neurosci.

[2] Kass RE, Ventura V, Brown EN (2005). Statistical issues in the analysis of neuronal data.. J Neurophysiol.

[3] Truccolo W, Eden UT, Fellow MR, Donoghue JP, Brown EN (2005). A point process framework for relating neural spiking activity for spiking history, neural ensemble and extrinsic covariate effects.. J Neurophysiol.

[4] Brown EN, Nguyen DP, Frank LM, Wilson MA, Solo V (2001). An analysis of neural receptive field plasticity by point process adaptive filtering.. Proc Natl Acad Sci U S A.

[5] Eden UT, Frank LM, Barbieri R, Solo V, Brown EN (2004). Dynamic analysis of neural encoding by point process adaptive filtering.. Neural Comput.

[6] Frank LM, Eden UT, Solo V, Wilson MA, Brown EN (2002). Contrasting patterns of receptive field plasticity in the hippocampus and the entorhinal cortex: an adaptive filtering approach.. J Neurosci.

[7] Frank LM, Stanley GB, Brown EN (2004). Hippocampal plasticity across multiple days of exposure to novel environments.. J Neurosci.

[8] Barbieri R, Frank LM, Nquyen DP, Quirk MC, Solo V (2004). Dynamic analyses of information encoding by neural ensembles.. Neural Comput.

[9] Brockwell AE, Rojas AL, Kass RE (2004). Recursive Bayesian decoding of motor cortical signals by particle filtering.. J Neurophysiol.

[10] Ergun A, Barbieri R, Eden UT, Wilson MA, Brown EN (2007). Construction of point process adaptive filter algorithms for neural systems using sequential Monte Carlo methods.. IEEE Trans Biomed Eng.

[11] Deneve S, Duhamel JR, Pouget A (2007). Optimal sensorimotor integration in recurrent cortical networks: a neural implementation of Kalman filters.. J Neurosci.

[12] Yu BM, Kemere C, Santhanam G, Afshar A, Ryu SI (2007). Mixture of trajectory models for neural decoding of goal directed movements.. J Neurophysiol.

[13] Shoham S, Paninski LM, Fellows MR, Hatsopoulos NG, Donoghue JP (2005). Statistical encoding model for a primary motor cortical brain-machine interface.. IEEE Trans Biomed Eng.

[14] Smith AC, Frank LM, Wirth S, Yanike M, Hu D (2004). Dynamic analysis of learning in behavioral experiments.. J Neurosci.

[15] Smith AC, Wirth S, Wendy AS, Brown EN (2007). Bayesian analysis of interleaved learning and response bias in behavioral experiments.. J Neurophysiol.

[16] Czanner G, Eden UT, Wirth S, Yanike M, Suzuki WA (2008). Analysis of between-trial and within-trial neural spiking dynamics.. J Neurophysiol.

[17] Srinivasan L, Brown EN (2007). A state-space framework for movement control to dynamic goals through brain-driven interfaces.. IEEE Trans Biomed Eng.

[18] Serruya MD, Hatsopoulos NG, Paninski L, Fellows MR, Donoghue JP (2002). Brain-machine interface: instant neural control of a movement signal.. Nature.

[19] Arabzadeh E, Zorzin E, Diamond ME (2005). Neuronal encoding of texture in the whisker sensory pathway.. PLoS Biol.

[20] Arabzadeh E, Panzeri S, Diamond ME (2004). Whisker vibration information carried by rat barrel cortex neurons.. J Neurosci.

[21] Vanrullen R, Guyonneau R, Thorpe S (2005). Spike times make sense.. Trends Neurosci.

[22] Kiani R, Esteky H, Tanaka K (2005). Differences in onset latency of macaque inferotemporal neural responses to primate and non-primate faces.. J Neurophysiol.

[23] Dayan P, Abbott LF (2001). Theoretical Neuroscience..

[24] Daley D, Vere-Jones D (2003). An Introduction to the Theory of Point Process, 2^nd^ ed.

[25] Brown EN, Barbieri R, Eden UT, Frank LM, Feng J (2003). Likelihood methods for neural data analysis.. Computational Neuroscience: A Comprehensive Approach.

[26] Singh R, Raj B (2004). Classification in likelihood spaces.. Technometrics.

[27] Salimpour Y, Soltanian-Zadeh H (2009). Particle filtering of point process observation..

[28] Jacobsen M (2005). Point Process Theory and Applications: Marked Point and Piecewise Deterministic Processes..

[29] Rolls ET, Deco G (2002). Computational Neuroscience of Vision..

[30] Butts DA (2003). How much information is associated with a particular stimulus?. Network.

[31] Brown EN, Barbieri R, Ventura V, Kass RE, Frank LM (2002). The time-rescaling theorem and its application to neural spike train data analysis.. Neural Comput.

[32] Cox TF, Cox MAA (2001). Multidimensional Scaling..

[33] Tamura H, Tanaka K (2001). Visual response properties of cells in the ventral and dorsal parts of the macaque inferotemporal cortex.. Cereb Cortex.

[34] Kiani R, Esteky H, Mirpour K, Tanaka K (2007). Object category structure in response patterns of neuronal population in monkey inferior temporal cortex.. J Neurophysiol.

[35] Hung CP, Kreiman G, Poggio T, DiCarlo JJ (2005). Fast readout of object identity from macaque inferior temporal cortex.. Science.

[36] Haxby JV, Gobbini MI, Furey ML, Ishai A, Schouten JL (2001). Distributed and overlapping representations of faces and objects in ventral temporal cortex.. Science.

[37] Paninski L (2004). Maximum likelihood estimation of cascade point-process neural encoding models.. Network.

[38] Paninski L, Pillow J, Simoncelli E (2004). Maximum likelihood estimation of a stochastic integrate-and-fire neural encoding model.. Neural Comput.

[39] Keat J, Reinagel P, Reid R, Meister M (2001). Predicting every spike: a model for the responses of visual neurons.. Neuron.

[40] Pillow J, Paninski L, Uzzell V, Simoncelli E, Chichilnisky E (2005). Prediction and decoding of retinal ganglion cell responses with a probabilistic spiking model.. J Neurosci.

[41] Quian Quiroga R, Panzeri S (2009). Extracting information from neural populations: information theory and decoding approaches.. Nat Rev Neurosci.

[42] Ince RA, Senatore R, Arabzadeh E, Montani F, Diamond ME (2010). Information-theoretic methods for studying population codes.. Neural Netw.

[43] Arabzadeh E, Petersen RS, Diamond ME (2003). Encoding of whisker vibration by rat barrel cortex neurons: implications for texture discrimination.. J Neurosci.

[44] von Heimendahl M, Itskov PM, Arabzadeh E, Diamond ME (2007). Neuronal activity in rat barrel cortex underlying texture discrimination.. PLoS Biol.

[45] Salimpour Y, Soltanian-Zadeh H (2009). Particle filtering of point process observation..

[46] Smith AC, Brown EN (2003). Estimating a state-space model from point process observations.. Neural Comput.

[47] Godsill SJ, Doucet A, West M (2004). Monte Carlo smoothing for nonlinear time series.. J Am Stat Assoc.

[48] Serruya MD, Hatsopoulos NG, Paninski L, Fellows MR, Donoghue JP (2002). Instant neural control of a movement signal.. Nature.

[49] Wessberg J, Stambaugh CR, Kralik JD, Beck PD, Laubach M (2000). Real-time prediction of hand trajectory by ensembles of cortical neurons in primates.. Nature.

[50] Taylor DM, Tillery SI, Schwartz AB (2002). Direct cortical control of 3D neuroprosthetic devices.. Science.

[51] Musallam S, Corneil BD, Greger B, Scherberger H, Andersen RA (2004). Cognitive control signals for neural prosthetics.. Science.

